# Interaction among inflammasome, PANoptosise, and innate immune cells in infection of influenza virus: Updated review

**DOI:** 10.1002/iid3.997

**Published:** 2023-09-26

**Authors:** Li Wei, Xufang Wang, Huifei Zhou

**Affiliations:** ^1^ Intensive Care Unit, Huzhou Third Municipal hospital The Affiliated hospital of Huzhou University Huzhou China

**Keywords:** inflammasome, influenza, innate immunity, PANoptosis

## Abstract

**Background:**

Influenza virus (IV) is a leading cause of respiratory tract infections, eliciting responses from key innate immune cells such as Macrophages (MQs), Neutrophils, and Dendritic Cells (DCs). These cells employ diverse mechanisms to combat IV, with Inflammasomes playing a pivotal role in viral infection control. Cellular death mechanisms, including Pyroptosis, Apoptosis, and Necroptosis (collectively called PANoptosis), significantly contribute to the innate immune response.

**Methods:**

In this updated review, we delve into the intricate relationship between PANoptosis and Inflammasomes within innate immune cells (MQs, Neutrophils, and DCs) during IV infections. We explore the strategies employed by IV to evade these immune defenses and the consequences of unchecked PANoptosis and inflammasome activation, including the potential development of severe complications such as cytokine storms and tissue damage.

**Results:**

Our analysis underscores the interplay between PANoptosis and Inflammasomes as a critical aspect of the innate immune response against IV. We provide insights into IV's various mechanisms to subvert these immune pathways and highlight the importance of understanding these interactions to develop effective antiviral medications.

**Conclusion:**

A comprehensive understanding of the dynamic interactions between PANoptosis, Inflammasomes, and IV is essential for advancing our knowledge of innate immune responses to viral infections. This knowledge will be invaluable in developing targeted antiviral therapies to combat IV and mitigate potential complications, including cytokine storms and tissue damage.

## Graphical abstract

Influenza virus causes infection in the respiratory tract. Innate immune cells are the key cells in combating iInfluenza virus infection through various mechanisms. Inflammasomes are the common mechanism in these immune cells that have a critical function in the elimination of viral infections. As well, cellular death mechanisms have also the protective role in innate immune responses. Pyroptosis, Apoptosis, and Necroptosis (PANoptosis) are the cellular death pathways to remove influenza infection.

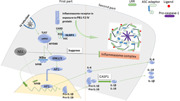

## 1 INTRODUCTION

The influenza virus (IV) is a member of the Orthomyxoviridae family with a genome containing a single strand of negative‐sense RNA. IV type A (IvA) has a genome structure consisting of 8 RNA strands that encode 11 proteins, including glycoproteins, hemagglutinin A (HA), and neuraminidase (NA).^1^ IvA has various subtypes, identified based on 16 HA and 9 NA strains responsible for vertebrate infections. IvA and IV type B (IvB) are two types of IV that can cause severe infections in humans.^2^ IvA can be divided into two subtypes, H1N1 and H3N2, while IvB has two different lineages, B/Victoria/2/87‐like (B/Vic) and B/Yamagata/16/88‐like (B/Yam).^3^ Other significant subtypes include H2N2, H5N1, H7N7, and H9N2, but H1N1 and H3N2 are the most common strains that cause human infections. IV infections usually occur during the last half of each year, especially during the cold season, and can affect individuals of any age. According to the World Health Organization (WHO), in 2018, seasonal IV infections accounted for 3–5 million cases of severe illness and approximately 290,000–650,000 respiratory deaths yearly.^4^, ^5^

Studies conducted in China have shown that influenza‐related deaths vary yearly. From 2007 to 2013, the average number of deaths due to IvA infection was 2375 per 100,000 individuals.^6^ The upper respiratory tract is where the virus typically colonizes and multiplies, leading to acute pulmonary inflammation. This inflammation is considered the most critical indicator of IvA infection.^7^, ^8^ The immune system consists of two major branches: innate and adaptive. Both play significant roles in fighting pathogens like bacteria, parasites, and viruses. Innate immunity is the first line of defense against IV. The critical components of innate immunity in combating IvA include Macrophages (MQs), Neutrophils, and Dendritic cells (DCs). Each component has specific mechanisms, such as respiratory burst, NETosis, pinocytosis, and Inflammasomes.^9^, ^10^, ^11^, ^12^ Neutrophils can perform phagocytosis, NETosis, and respiratory burst in response to IV. MQs use phagocytosis, pinocytosis, and respiratory burst, while DCs perform pinocytosis and phagocytosis to counteract IV.^12^, ^13^, ^14^

According to studies, inflammasome is an important immune defense mechanism against IvA that helps to clear IvA infection. Inflammasomes are special complexes of the innate immune system, acting as receptors and sensors.^15^, ^16^ They regulate the activation of the caspase family and stimulate inflammation when exposed to IvA or self‐proteins. Inflammasomes are closely linked to cellular death events, which can help defend against microbial pathogens such as IV.^17^, ^18^ Apoptosis, necrosis (a key component of inflammatory procedures), pyroptosis, and necroptosis (a regulated form of apoptosis and necrosis) all play a role in maintaining tissue homeostasis, embryonic development, combating cancers, and autoimmune disorders, and assisting immune responses against infections.

The biological process of programmed cell death is known as apoptosis. It plays a crucial role in morphogenesis, cell turnover, and removing harmful cells. Balanced apoptosis is necessary for maintaining good health. Activating effector proteases called caspases lead to cellular disassembly and ultimately cause cell death. Apoptosis can occur through various pathways, including mitochondria‐independent and dependent routes. It differs from necrosis, which results from the failure to control cellular homeostasis after damage. Unlike necrosis, apoptosis is a highly regulated process induced by a specific stimulus that does not release inflammatory mediators. Pro‐inflammatory programmed cell death differs from apoptosis, despite its similarities with pyroptosis. The key difference between apoptosis and pyroptosis is that the former is noninflammatory, while the latter is pro‐inflammatory. Necroptosis is a caspase‐independent form of cell death that can be triggered by treatment with TNF. It is considered to be a regulated form of necrosis.^19^, ^20^

The term PANoptosis refers to a trio of cell death mechanisms: Pyroptosis, which is inflammation‐related programmed cell death; Apoptosis, which is a complex molecular process that leads to cell death; and Necroptosis, which is a programmed form of necrosis accompanied by inflammation^20^, ^21^ (Box 1). However, the exact interaction between the IV, inflammasome, PANoptosis, and other innate immune cell defense mechanisms is not fully understood.

Box 1. **Highlighted points in Innate immunity, inflammasomes, and PANoptosis in IV infection**

**Table:** 

MQs, NEs, and DCs have various mechanisms to counteract the IV infection in which the Inflammasome is the common mechanism in these cells. Apoptosis is a cellular death pathway that occurs in infected cells with IV. Inflammasomes can form following innate immune sensing of RNA‐IV. Pyroptosis is an immunological death mechanism which is along with the production of pro‐inflammatory cytokines (IL‐1β, IL‐18) and lysis of infected cells with IV. Necroptosis is an inflammatory mechanism independent of Caspases and is activated by specific proteins of IV. Caspases are the key players in apoptosis and pyroptosis mechanisms. RIPK1, RIPK3, and MLKL have a major role in the necroptosis pathway.

The immune system has a branch called adaptive immunity, composed of two main parts: cellular (including T cells, T‐helper, regulatory T cells, and cytotoxic T cells) and humoral (consisting of B cells and antibodies). These components play a crucial role in fighting against pathogens.^22^

The objective of this study is to provide a summary of the most recent research findings and uncover the interrelated connections within the complex network.

## 2 APOPTOSIS AND INFLAMMASOME COMPLEX: JUNCTION POINT

The caspases family including a group of cysteine‐dependent aspartate is responsible for apoptosis induction. Generally, caspases are subdivided into initiator (caspase‐8, −9, and −10) and executioner (caspase‐3, −6, and −7) that amplifies the initial signal and induces cell death respectively. As well, extrinsic and intrinsic pathway is another classification of apoptosis in which death receptors (TNFR) are activated and belong to the extrinsic pathway (caspase‐8 is the axis molecule). The intrinsic pathway is activated following the release of cytochrome C from mitochondria and finally apoptosome is formed (caspase‐9 is the key molecule).^23^

**Figure 1:**
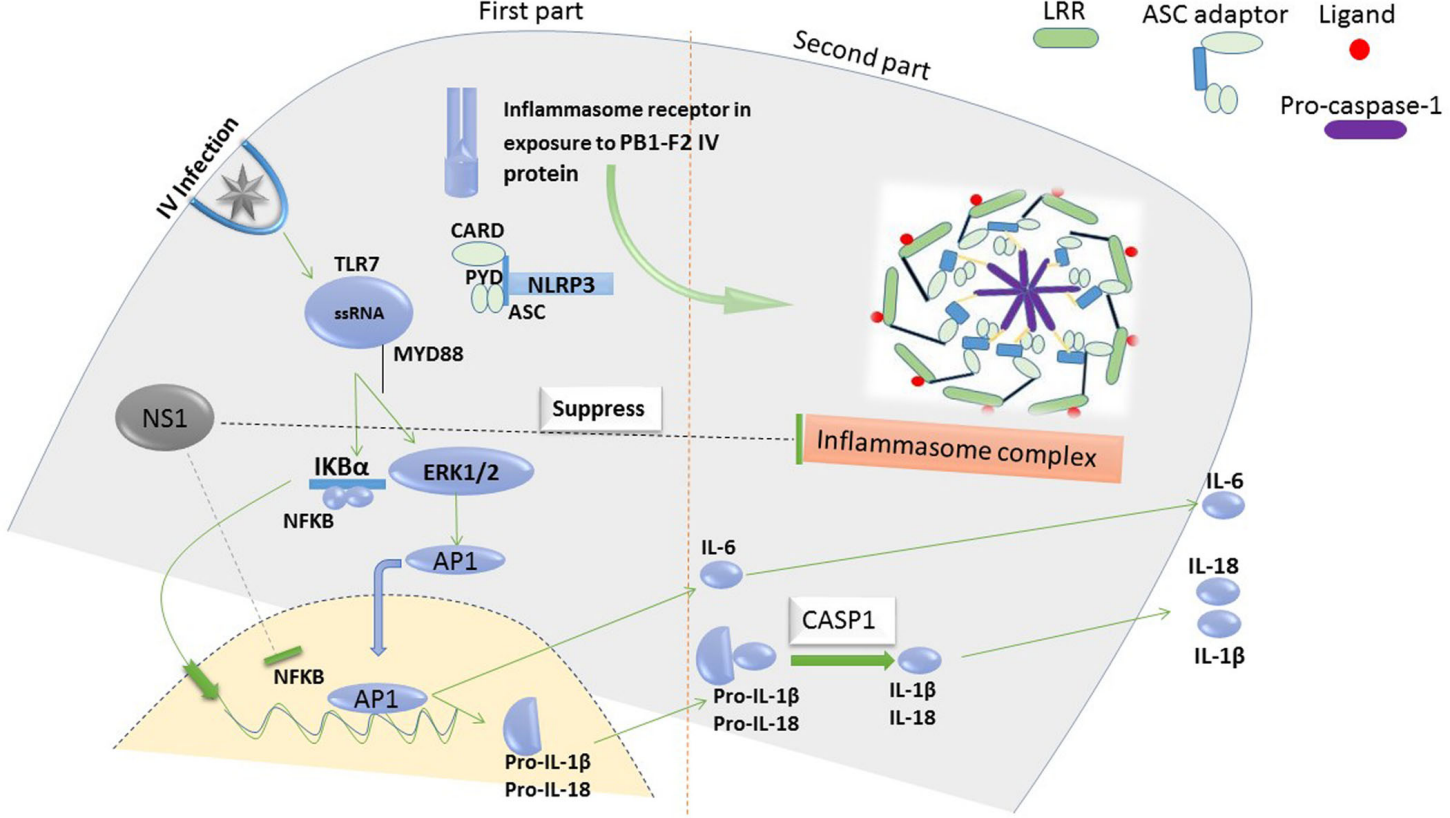
A map‐way of inflammasome activation following sense of RNA virus. The initial phase is related to the activation of NFκB, AP1, and Caspase‐1 then with increasing sensitivity of inflammasome components. As well, IL‐1β and IL‐18 are produced in inactivation form. The second part is attributed to NLRP3 activation and recruitment of adaptor proteins (ASC and CARD domain) and caspase‐1. At least, activated inflammasome assembly leads to making a pore following in pyroptosis mechanism in target host cells infected with IV.

Activation of inflammasomes is rely on caspase‐1 activity and secretion of IL‐1β and IL‐18 pro‐inflammatory cytokines. Caspase‐1 and these inflammatory cytokines can lead to induction of Gasdermin D (GSDMD) which in turn pore formation occur in the cellular membrane of the target.^24^, ^25^ Two signals are required to activate inflammasome: (1) The first signal is related to the involvement of pattern recognition receptor (PRR) and activation of NFƘB transcription factor by Toll‐like receptors (TLRs), Tumor necrosis factor receptor (TNFR) an IL‐1 receptor which results in two important events (1.1) raising the sensitivity of the sensor through the upregulated level of inflammasome components and then (1.2) production of the inactive form of pro‐IL‐1β and pro‐IL‐18 cytokines. (2) NLRP3 is considered as a second signal which is activated by damage to host cells. Activated NLRP3 recruits ASC adaptor, proapoptotic protein containing CARD domain (Caspase Recruitment Domains; a homotypic protein identified as death fold), and then caspase‐1 is recruited. Also, activated inflammasome complex leads to destroying the IV‐infected cellular membrane.^26^ Based on the reports, apoptosis can be converted into pyroptotic cell death through activation of GSDMD; however, it is not clear if this event could be performed on the contrary way or not. This finding has been confirmed via the deletion of apoptotic caspases which has no effect on the progression of pyroptosis^27^, ^28^ (Figure 1). Collectively, although caspases particularly caspase‐1 is responsible for the pyroptosis mechanism directly, caspase‐8 could be deemed as the junction point between the inflammasome (pyroptosis) and apoptosis pathway.

## 3 INTERCONNECTION OF PANOPTOSIS, INNATE IMMUNE RESPONSES, AND INFLAMMASOMES IN IV INFECTION: FIGHT OR EVADE?

Regarding the mentioned earlier, there are also various determinative cellular mechanisms including apoptosis, necrosis, pyroptosis, and necroptosis which are critical for IV infection. These pieces of cellular evidence are highly observed in IV infections which have significant connection to the inflammasome and thus innate immune cells such as macrophages, neutrophils, and Dendritic cells. Pyroptosis and necroptosis are regulated forms of apoptosis and necrosis respectively which are effective in the elimination or even progression and development of IV infections.^29^, ^30^, ^31^, ^32^ Mixed lineage kinase domain‐like protein (MLKL) and receptor‐interacting protein kinase‐3 (RIPK3) are the key receptors of necroptosis.^33^, ^34^ In this respect, some scientists have reported murine protein called Z‐DNA/RNA binding protein 1 (ZBP1; also known as DAI/DLM‐1) as a considerable parameter of pyroptosis, necroptosis, and apoptosis mechanisms could be stimulated by IvA. They showed that ZBP1 can interact with RIPK3 and thereby manage Influenza development and progression.^35^, ^36^, ^37^

Immune responses are usually initiated by Pattern Recognition Receptors (PRRs) against micro‐organisms.

PRR is a category of receptors that is able to directly detect the specific molecular structures on the surface of apoptotic host cells, pathogens, and damaged cells. PRRs connect between nonspecific immunity and specific immunity. Through the detection and binding of ligands, PRRs can produce nonspecific responses of anti‐infection, antitumor, and even other immunoprotective reactions. PRRs can be classified into the five types based on protein domain homology: Toll‐like receptors (TLRs), nucleotide oligomerization domain (NOD)‐like receptors (NLRs), C‐type lectin receptors (CLRs), retinoic acid‐inducible gene‐I (RIG‐I)‐like receptors (RLRs), and absent in melanoma‐2 (AIM2)‐like receptors (ALRs).^38^, ^39^ As mentioned in the previous section, inflammasomes can be formed when exposed to sensor molecules and IV or other microbial pathogenic and thus a complex of NLRs and ALRs with ASC (CARD) and also pro‐caspase‐1 come together and make a multiprotein substance that is able to produce IL1β inflammatory cytokine. NLRs and AIM2 as the receptors of inflammasome can recognize their own ligands that is, the Naip‐NLRC4 inflammasome identifies bacterial proteins The NAIP‐NLRC4 inflammasomes respond to a broad spectrum of pathogens, such as Shigella, Salmonella, and Legionella, in which NAIPs display high ligand specificity. As well, binding of DNA to AIM2 induces its oligomerization and recruitment of ASC through its pyrin domain, resulting in activation of caspase 1 as in the case of the NLRs. Activation of Inflammasome by AIM2 is responsible for IL‐1β release following infection from various DNA viruses such as vaccinia virus and adenovirus.^38^, ^40^

NLRP1 can identify anthrax lethal toxin (ALT) and NLRP3 recognizes bacterial toxins, especially various proteins of IV and other stimuli such as ATP. DNA and IV infections are recognized by the AIM2 receptor. In addition, NLRP6, 9, and 12, PIRYN, and other inflammasome receptors are observed to initiate inflammasome assembly. Inflammasomes are the common defense mechanism in MQs, NE, and DCs as innate immune cells.^38^, ^40^, ^41^

Further, NLRs, RLRs, and TLRs, as PRRs family, are identified to cooperate for early detection of IvA and induce host responses which are regarded as protective, aid in removing infection, and can promote disease burden when responses are considered as further and prolonged. HA, as an IvA glycoprotein, can attach to sialic acid expressed by a cell surface receptor. Therefore, endosome is formed and its acidification is initiated. Finally, initial signals of the inflammasome pathway including TLR3, TLR7, and RIG‐1 are triggered following sensed IVA by PRRs. TLR7 binds to ssRNA in the endosomal membrane, while TLR3 is activated by dsRNA species generated during IvA infection. RIG‐I detects cytosolic ssRNA and triggers inflammatory and antiviral responses by attaching with MAVS via the transcriptional factors NF‐κB and IRF. Furthermore, sensing viral RNA and subsequent activation of the NLRP3 inflammasome is reported to be a critical part of the host defense against IvA infection. The activation of the NLRP3 inflammasome during sublethal IV infection performs a substantial function in limiting lung damage resulting from infection.^26^, ^42^

NLRP1 can be activated by guramyl dipeptide, while NLRC4 is activated by bacterial flagellin including Salmonella typhimurium and Legionella pneumophila or some bacterial proteins of gram‐negative bacteria. The NLRP1 and NLRC4 inflammasome complex speck forms through the NLR CARD, which can assemble by direct CARD‐CARD interaction with pro caspase‐1 or indirect interaction with ASC.^43^

Excessive inflammation generated by immune responses can lead to cytokine storms which are regarded as responsible for clinical complication known as acute respiratory distress syndrome (ARDS). ARDS is triggered by a further immune response rather than the viral load. Overproduction of inflammatory mediators and cytokines such as inflammasome complexes, IL‐6, IL‐1β, TNF‐α, and the like leads to lung fibrosis in affected patients. Thus, using ventilators as initial actions plays a critical role in saving the life of the aforementioned patients.^44^, ^45^

### 3.1 Macrophages(MQs) and IV

MQs are important cells in the body's defense against pathogens. They have receptors on their surface called TLRs, NLRs, and ALRs. There are different types of MQs, including M1 and M2. M1 MQs produce inflammatory cytokines and are responsible for phagocytizing intracellular microbes. M2 MQs, on the other hand, have anti‐inflammatory properties and are involved in tissue repair. M2 MQs are further divided into M2a, M2b, M2c, and M2d subgroups. IL‐4 and IL‐13 activate M2a, whereas M2b responds to lipopolysaccharides (LPS) and immune complexes. M2c is activated by transforming growth factor beta (TGF‐β) and glucocorticoids, while M2d responds to adenosines and IL‐6 cytokines. The exact MQ phenotype depends on the environment in which they are found. By reprogramming transcription, factors like cytokines can determine whether MQs have an M1 or M2 phenotype. The M2 phenotype is favored in the lung due to its anti‐inflammatory properties (M2 > M1 ratio).^46^, ^47^, ^48^

When MQs are infected with IV, they can limit virus production through their immune functions. While some studies suggest that IV can evade MQ functions,^49^, ^50^ another study found that mice without MQs had higher viral loads, highlighting the important role of MQs in defending against IV.^51^, ^52^ IV typically affects lung tissue and respiratory tracts through inflammatory responses, and lung tissue remodeling is crucial to maintain proper functioning. However, some respiratory disorders, such as asthma, can result. IV can activate two enzymes in MQs, collagenases, and metalloproteases, damaging lung tissue and potentially leading to death.^53^ Excessive inflammation caused by immune responses can result in cytokine storms and acute respiratory distress syndrome (ARDS), triggered by further immune responses rather than viral load. Overproduction of inflammatory mediators and cytokines, such as inflammasome complexes, IL‐6, IL‐1β, and TNF‐α, can also lead to lung fibrosis in affected patients. Therefore, using ventilators as an initial action is crucial for saving the lives of these patients.^44^, ^45^

Although macrophages produce a variety of pro‐inflammatory cytokines (IFN‐β, IL‐β, IL‐18, and TNF‐α) when exposed to IV, the level of these cytokines is largely influenced by the induction of apoptosis in alveolar macrophages (AMs).^54^, ^55^ When NLRs interact with PB1‐F2 (a proapoptotic protein of IV), they can shield AMs from IV‐induced apoptosis, which leads to an increase in the production of IFN‐β.^56^

In a study conducted by Joanna Jaworska and colleagues they examined the immunity of MQs in mice infected with IV (influenza virus). Their research found that NLRX1, a member of the NLR family, has antiviral properties that boost the innate immune response, improve immunity against IV, promote IFN‐β signaling, and increase survival rates of infected MQs. The study also discovered that NLRX1 regulates mitochondrial‐induced apoptosis by interacting with the PB1‐F2 proapoptotic protein.^57^, ^58^

We have discovered that IFN‐β can protect AMs from IV‐induced apoptosis. Additionally, research has suggested a correlation between IFN‐β and PGE2 levels during viral infections. Specifically, increased PGE2 levels can hinder IFN‐β production and decrease viral antigen presentation to T cells in AMQs infected with IV. Conversely, mice without PGE2 had a higher survival rate when exposed to acute IV infection.^59^

In the context of IV infections, the IL‐6 cytokine plays a significant role in the immunopathology of MQs. It also promotes the recruitment of monocytes (Ly6 positive) and the maturation of regulatory T cells (Tregs) through IL‐27 signaling.^60^ AMs in mice infected with IV have been found to express TNF‐related apoptosis‐inducing ligand (TRAIL), resulting in apoptosis in epithelial cells. This is believed to be a preemptive activity of innate immunity to reduce susceptibility to IV infection.^61^, ^62^ *Csf2* ^−/−^ mice have demonstrated increased morbidity in response to IV infection due to the impaired function of AMs, while the population and function of cytotoxic T cells remain intact.^63^ These findings suggest that AMs have a significant relationship with adaptive immunity (humoral and cellular immunity) in clearing IV infections. AMs also express TGFβR and CD200R receptors that convey preventive signals to decrease the antiviral functions in AMs.^64^, ^65^ During an IV infection, AMs exhibit different phenotypes and functions. As necessary, MQs can provide an inflammatory response and perform tissue repair.

A study by Sun and colleagues revealed that AMs expressing PPAR‐γ, a receptor can suppress acute IV infection in mice.^66^ It was observed that the expression of PPAR‐γ decreases in AMs infected with IV. PPAR‐γ is critical for the antiviral response of AMs and can modulate cell growth, differentiation, and proliferation of genes. The reduction of PPAR‐γ leads to downregulating IFN‐type I signaling, resulting in a more destructive IV infection. Interestingly, a study on Macaques with lethal IV infection showed a significant loss of AMs and the recruitment of activated interstitial macrophages^CCR2+ CX3CR1+^ (IMQs) in the infected respiratory tract.^67^ Additionally, an abundant population of MQs in the lower respiratory tract plays a key role in tissue remodeling and eliminating IV after viral infection.^47^, ^68^ Overall, MQs have essential functions during IV infection.

### 3.2 Neutrophils (NEs) and IV

The NE is a vital cell that serves important functions and can be recruited by various mediators, including CXCL1, CXCL2, and CXCL5. In cases of IV infection, the cytokine IL‐8 is the most effective agent for bringing NEs from the blood vessels into the lungs. NEs can help contain IV infections, but an excessive number can cause inflammatory damage to the infected lung tissue.^69^, ^70^

NEs have a protective role during viral infections through multiple defense mechanisms, including

1. *Phagocytosis:* NEs perform phagocytosis, a crucial function involving engulfing viruses and infected cells. Although NEs use antimicrobial agents and enzymes to eliminate microorganisms after IV phagocytosis, reports suggest that NEs' phagocytosis can sometimes assist viruses in evading the immune system.^13^, ^71^
2. *Degranulation:* NEs release granules containing lactoferrin, myeloperoxidase (MPO), H_2_O_2_, elastase, cathepsin G, proteinase 3, NSP4, and other substances during viral infections. This process is called degranulation.^72^ Researchers, such as Superti et al.^73^ have found that bovine lactoferrin can suppress IaV infection by inhibiting the fusogenic activity of the viral HA.
3. *Production of cytokines, antimicrobial substances:* When someone has a viral infection, their body releases pro‐inflammatory mediators like cytokines (IFNs, IL‐8, TNF‐α, and IL‐6) which can suppress the infection's replication and progression. This can also be a chemotactic function to attract and activate more NEs. Additionally, NEs release MMP9 to remove the infection and increase the NE population's infiltration into the lung by decomposing the extracellular matrix.^71^, ^74^
4. *Respiratory burst*: NEs utilize NADPH oxidase to catalyze the reactive oxygen species (ROS) against IV. NADPH oxidase system is applied to control virus spreading via the degrading of chemical groups and the substances necessary for IV replication.^75^, ^76^
5. *Neutrophil extracellular traps (NETs):* NETs are composed of the unique structure of chromatin covered by cytoplasmic proteins (histones, defensin, MPO, elastase) and antimicrobial particles. Studies indicated the antiviral function of NETs through trapping the viral particles and killing the virus with a high dose of defensin and MOP elements.^71^, ^77^

### 3.3 Dendritic cells (DCs) and IV

DCs are a type of innate immune cell with antiviral defense mechanisms. These professional antigen‐presenting cells directly connect the innate and adaptive immune systems, displaying a key phenotype in the upper respiratory tract. When activated by counteracting IV infection, CD103+ DCs migrate to the lymph node and use cross‐presentation to present viral antigens to CD8+ T cells.^78^ Studies have shown that depletion of these DCs in mice resulted in a more severe acute phase of infection than in mice with healthy CD103+ DCs. Therefore, DCs play an important role in combating IV infections.^79^ Recent research has identified the PA‐X protein‐derived H1N1 as a mechanism for IV to evade immune responses by interfering with the maturation of DCs, a crucial step in viral antigen presentation. Additionally, PA‐X can inhibit the production of IFN‐I through the RIG‐I‐mediated signaling pathway.^80^, ^81^

Totally, it has been determined that innate immune cells such as MQs, Neutrophils, and DCs play a crucial role in eliminating IV infections through specific immune responses. However, studies have shown that IV has multiple evade mechanisms that allow it to escape innate immunity. To propagate and amplify an infection, IV needs to evade the innate immune response.^82^, ^83^

Generally, it has been believed that IV can accelerate the speed of its replication to evade from cellular death pathway.^83^ On the other hand, NS1, an Influenza protein, can limit the assembly of NLRP3 inflammasome through the suppression of recruitment of the downstream adaptor proteins. Consequently, IL‐1 β and self‐oligomerization of NLRP3 is impaired. As a result, NS1 has a suppressive role in the pyroptosis pathway.^84^ Some studies have been carried out about the inductive effect of NS1 on the necroptosis pathway in MQs infected with IvA through interacting with MLKL. The outcome of this interaction is the increment of oligomerization of MLKL, and eventually, IL‐1β increases; hence, the necroptosis is activated.^11^, ^85^ Based on this finding, NS1 has opposite effects on pyroptosis and necroptosis. Along with this, NS1 can hinder NFƘB activation by targeting IKK‐β. Also, it can directly attach to PKR that has antiviral activities in innate immune responses and then modulate PKR antiviral function. NS1 also can inhibit the RIG‐1 signaling cascade.^86^ Additionally, PB1‐F2 can activate NLRP3 inflammasome over and over and surprisingly impair it due to having various secondary structures of the spliceosome.^87^ As well, PB1‐F2 is considered the antagonist of IFN‐I by targeting DEAD‐box helicase 3 X‐linked (DDX3), a host protein with antiviral activity.^86^ Zhang et al.^88^ indicated that NS2 of H1N1 has a suppressive function on IFN‐β production through targeting IRF7 (interferon regulatory factor 7). Thereby, IV can apply several mechanisms to evade innate immune responses.

As outlined above, the innate immunity of MQs, DCs, and NE partly depends on inflammasome activity. Therefore, IvA‐infected MQs and DCs produce IL18 and IL‐1β pro‐inflammatory cytokines following inflammasome activation. NLRP3, the key component of inflammasome function in mentioned immune cells, is well known. NLRP3 expression and ASC and caspase‐1 are needed to cleave pro caspase‐1 to secrete mature IL‐1β following IV infection.^89^ NLRC4 is another NLR that can form an inflammasome without the contribution of NLRP3. However, Allen et al.^26^ have reported that NLRP3^–/–^ mice could not survive after IV infection while NLRC4 inflammasome was intact. The results of this study indicated a pivotal role of NLRP3 in eliminating IV infections. The signaling of IL‐1β mediated by NLRP3 inflammasome can also recruit NEs for virus clearance and host survival in IV infection.^90^ In this regard, Momoeta and colleagues evaluated the infiltration of NEs and MQ into IV‐infected mice lungs through the interaction between ZBP1 (Z‐DNA binding protein 1) and IL‐1α. Like IL‐1β, IL‐1α could initiate infiltration and influx of neutrophils to the lung during IV infection. Studies have demonstrated that inflammasomes could damage the lung tissue following acute inflammatory reactions by NLRP3 and RIP3 pathways. As well, RIP3 and its upstream factor ZBP1 were shown to control not only necroptotic cell death but also NLRP3 activation and IL‐1β secretion. They showed that ZBP1 can modulate neutrophil influx and NET formation into the lung via managing necroptotic cell death and controlling NLRP3 activation and IL‐1β release during IV infection.^35^, ^91^

## 4 INFLAMMASOME SIGNALING PATHWAYS IN IV INFECTION

To review the signaling pathway, we must go back a little Inevitably.

There are two pathways for inflammasome activation: (1) canonical and (2) noncanonical.^92^

In the canonical pathway, there have two important signals for inflammasome triggering in which NLRP3 has the key‐axis role. TNF‐α, Polyriboinosinic polyribocytidylic acid (Poly (I:C), Pam3CSK4 (Toll‐like receptor‐2 agonist), and Lipopolysaccharide (LPS) are the initial signals for priming and activating of NF‐ƘB (nuclear factor‐kappa B) and ERK (extracellular signal‐regulated kinase) signaling pathways. Actually, upregulation of gene expression of Inflammasome components, that is, NLRP3 and IL‐1β, are the most powerful parameters for priming and activating the downstream inflammatory pathways. Before inflammasome assembly, NLRP3 activation is tightly modulated by posttranslational modifications such as phosphorylation, ubiquitination, and sumoylation. These modifications prevent from auto‐activation of the inflammasome in a competent manner.^27^

Various stimuli such as mitochondria dysfunction, K+ efflux, and trans‐Golgi disassembly induce specific stress patterns which NLRP3 almost touches. However, some pathways called noncanonical have been proposed, which occur during bacterial and viral infections, caspase‐4 and caspase‐5 in humans and caspase‐11 for mice. Finally, when GSDMD is cleaved, and the pyroptosis mechanism begins, NLRP3 inflammasome is activated. Particularly in IV infection, NLRP3 inflammasome is activated following capturing RNA of IV (RNA‐IV).^93^ Generally, RNA‐IV reinforces this process via lysosomal maturation or ROS. M2, as some IV protein, can facilitate inflammasome activation by the M2 ion channel.

As mentioned earlier, PB1‐F2 also activates inflammasome. While the NS1 protein of IV suppresses inflammasome by targeting NF‐ƘB activation. MEK1/2‐ERK1/2 signaling pathway includes cFos and c‐Jun as transcription factors that are important in regulating gene expression.

In this regard, Wan and colleagues indicated that ERK/AP‐1 signaling pathway increases the transcription of pro‐IL‐1β to facilitate the activation of NLRP3 inflammasome upon IV infection. The signaling pathway of ERK/AP‐1 confronts the IV via activating inflammasome.^39^

## 5 PANOPTOSIS IN IV INFECTION

Pyroptosis and its relationship with inflammasome were described before. PANoptosis, its interaction with inflammasome, and its interconnection with IV infection are explained here.

Dead cells take the appearance of a swollen balloon or rupture of the plasma membrane in exposure to TNF‐α as the key cytokine in the necroptosis procedure. Based on the studies, apoptosis inhibition led to necroptosis. The above‐mentioned event was reported in immune responses against viral infections. Current studies indicate that TNF‐α and IFN‐γ cytokines play an undeniable antiviral role in necroptosis procedure.^94^, ^95^ NEs and MQs can drive necroptosis in the condition of viral infections. Some differences are observed in the molecular mechanisms, although pyroptosis and necroptosis are considered lytic.

As indicated, pyroptosis acts as an outcome of inflammasome driving the downstream pathway caspase‐1 on GSDMD, leading to membrane rupture via the osmotic pressure.^96^ In addition, the necroptosis mechanism occurs through a complex of three major proteins (necroptosis), including MLKL, receptor‐interacting serine/threonine‐protein kinase 1 (RIPK1), and RIPK3.^35^ Phosphorylation is regarded as the key step of the cascade pathway in necroptosis, in which autophosphorylation of RIPK1 leads to the phosphorylation and activation of RIPK3. The phosphorylated‐activated RIPK3‐p recruits phosphorylate MLKL to oligomerize and make the pore‐forming composition which migrates to the cell membrane and leads to pores in the same method as the pyroptosis mechanism. Necroptosis is simulated through an independent mechanism of RIPK1. However, RIPK3 complexes with TRIF, DAI, or TRADD lead to MLKL activation and necroptosis. In addition, RIPK3 can be activated directly by the ASC‐NLRP3 inflammasome in an independent method of RIPK1, leading to MLKL activation and necroptosis.^97^, ^98^ PANoptosis is considered an effective mechanism to eliminate IV infection. For instance, mice deficient in IAP, a master regulator of programmed cell death, exhibited an increased mortality rate, while the immune function was regarded as intact.^99^, ^100^ As indicated, PANoptosis benefits from the same regulated proteins‐and‐signaling pathway, considered the most critical instrument in counteracting microbial infections, especially IV infections. Necroptosis and pyroptosis belong to two pathways of programmed cell death, creating a pore in the membrane of IV‐infected cells by executive proteins such as MLKL and GSDMD, respectively (Table 1).

**Table 1:** Comparative properties of PANoptosis.

Characteristics	Pyroptosis	Apoptosis	Necroptosis
Predisposing parameter	Pathological irritation	Physiological condition	Pathological irritation
Apoptotic body	No	Yes	No
Membrane morphology	Flatting	Bulge	Explosion
Chromatin condensation	Yes	Yes	Mild (partly)
Nucleic acid fragmentation	Yes	Yes	NO
Membrane intact	Raptured	Integrity	Raptured
Swollen organelle	Yes	No	Yes
Caspase activation	Yes	Yes	No
ROS production	Yes	Yes	Yes
MLKL, RIP3, RIP1	No	No	Yes
Procedure of cell death	Casp‐8, NLRP3	Casp‐3,7	ROS, MLKL, RIPK3
Inflammatory nature	Yes	No	Yes
Pore formation	Yes	No	Yes
Osmotic lysis	Yes	No	Yes

## 6 NETWORK OF THE INFLAMMASOME CELLULAR DEATH PATHWAYS IN IV INFECTION

Apoptosis is regarded as a cell defense mechanism in exposure to pathogenic micro‐organisms. Based on the studies, viruses can subvert apoptosis in the host cells.^17^ Viral replication can be limited in the early stage of apoptosis, while late apoptosis facilitates the shedding and release of viruses. The Intrinsic and extrinsic pathway of apoptosis can be induced by some proteins of IvA such as ribonucleoprotein complex (vRNP), NA, and HA which can interact with host proteins. Accordingly, the membrane of mitochondria undergoes perturbation and apoptosis signaling pathway is activated.^11^ For example, vRNPs can engage with a DNA‐dependent activator of interferon (IFN)‐regulatory factor (DAI) which stimulates caspase‐8 and caspase‐3, respectively, resulting in initiating apoptosis.^101^ As indicated, DAI or ZBP1 (specifically Zα domain) acts as a cytosolic DNA sensor and can participate in inducing the apoptosis while IvA infection is initiated. In addition, RIPK3 is recruited by DAI to stimulate the downstream signaling pathway through RHIM (RIPK homotypic interaction motif) domains. Therefore, the axis way of DAI/RIPK3/caspase plays a critical role in apoptosis induction.^102^

NS1 is considered as another IvA protein which increases viral replication by suppressing IFN‐I cytokine and exhibits a regulatory function in apoptosis induction through an unknown mechanism. The NS1 protein can trigger necroptosis and promote oligomerization of MLKL and translocation of the plasma membrane. Thus, excessive inflammation occurs the following necroptosis.^85^

Other specific proteins related to IvA such as HA and vRNP indicated an interaction with DAI and induction of necroptosis by recruiting RIPK3 and activating MLKL.^95^

PANoptosis are induced through the interaction of DAI with RIPK3/MLKL (Necroptosis), NLRP3/Caspase‐1 (Pyroptosis), and RIPK1/FADD/Caspase‐8 for triggering the apoptosis signaling pathway.^85^, ^95^

**Figure 2:**
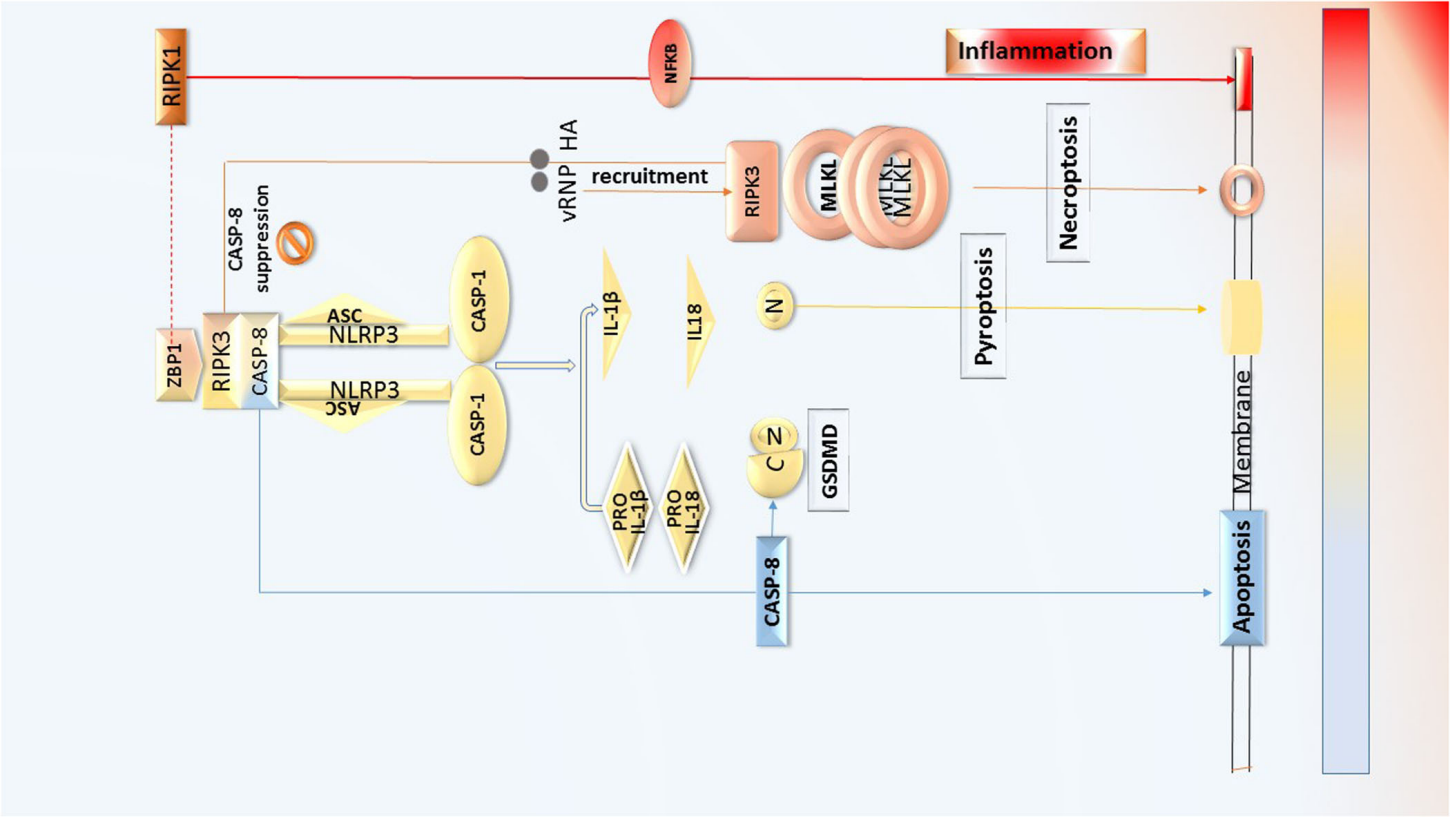
PANoptosis pathways in host cells exposed to IV proteins. Apoptosis is general cellular death pathway in which Caspases are key players in this mechanism. Apoptosis occurs without an inflammation and the cell membrane remains intact. On the other hand, Pyroptosis and necroptosis are immunological death pathways that cause inflammation. ZBP1, RIPK3, and NLRP3 and caspases are the major molecules in pyroptosis and finally pro‐inflammatory cytokines (IL‐1β and L‐18) are secreted through created the pore with the effect of GSDMD and Caspase‐1. Necroptosis occur independently of Caspases. RIPK1, RIPK3, and MLKL are the main proteins in this inflammatory mechanism in which TNF‐α is the executioner cytokine to make osmotic lysis in the target cells. Some IV proteins such as NS1, vRNP, and HA stimulate necroptosis and over‐inflammatory responses could occur. (Color: gradient of red and orange is related to inflammation followed by necroptosis. The yellow color is related to fewer inflammatory reactions in the pyroptosis mechanism and the blue color belongs to apoptosis without inflammation). HA, hemagglutinin A.

MQs and DCs which can promote NLRP3 inflammasome as two crucial innate immune cells regulate downstream transcription factors such as RIPK3, NF‐kB, and MAP kinase pathways to induce chemokines and cytokines. Some studies reported the activator function of RIPK3 in forming the inflammasomes directly in which such complex can be assembled with FADD/TRIF/RIPK1/caspase‐8. NFƘB expression is upregulated through mediating MLKL‐related NLRP3 activation in bystander cells. MLKL is transferred into the plasma membrane which leads to the efflux of potassium, resulting in activating NLRP3 intrinsically (Figure 2).^21^, ^82^, ^103^

To prevent excessive inflammatory reactions, MQs phagocyte apoptotic bodies are infected with IV which is almost found in alveolar routes and lung tissue. However, pyroptosis and necroptosis are observed, along with the inflammation due to releasing necrotic cells and applying specific mechanisms, especially inflammasomes. Therefore, an inflammatory condition in the respiratory tract worsens due to the consistent secretion of pro‐inflammatory cytokines (IL‐1β, IL‐18, and TNF‐α) through pyroptosis and necroptosis mechanisms. Finally, further inflammation generated within the entire respiratory tract can damage the integrity of lung parenchyma and epithelial cells.^104^, ^105^ TLR3/4 is upregulated during IV infection. Then, RIPK3 is recruited and downstream molecules such as NFƘB, MYD88, and TRIF are activated, resulting in triggering necroptosis pathway.^106^, ^107^

Based on the studies, RIPK3 can induce necroptosis without inhibiting the caspase‐8. RIPK3 can be activated directly by replicating IvA, Then, necrosome complex (RIPK1/MLKL/FADD) is stimulated. Kesavardhana and colleagues assessed the antiviral function of DDX3X during IvA infection and reported that the host protein DDX3X in interplay with NLRP3 inflammasome plays a crucial role in organizing a multifaceted antiviral innate response during IvA infection. DDX3X can activate the NLRP3 inflammasome in response to IvA. However, NS1 can interfere in the antiviral function of DDX3X and IFN‐I signaling, resulting in impairing the formation of stress granules. Thus, NS1 can hinder DDX3X activity without DDX3X, leading to death of IvA‐infected mice. However, DDX3X function appears to be intact in the absence of NS1 although cooperation of DDX3X with NLRP3 inflammasome can counteract NS1 protein seriously.^108^

In addition, Wan and colleagues evaluated the role of Gasdermin E (GSDME) in H7N9 IV infection and found that GSDME is regarded as responsible for cytokine storm and pyroptosis in epithelial cells in mice lungs infected with H7N9 IV. Mice infected with H7N9 virus survived in the absence of GSDME (knockout). Finally, Wan et al.^109^ argued that GSDME is considered as the major agent in exacerbating the inflammatory responses in IV infection. Caspase‐3 activates GSDME by cleaving GSDME to produce its N‐fragments which can create a pore in the plasma membrane in the pyroptosis mechanism (Table 2).

**Table 2:** Comparison of mode of action of specific proteins of influenza virus and some host proteins.

List	Influenza virus	Host protein	Mode of action
PB1‐F2	✓		Increase apoptosis in host cells
M2	✓		Activates inflammasome
NS1	✓		Suppresses inflammasome by targeting NF‐ƘB activation
PA‐X	✓		Cause to evade IV from immune responses by interfering in the maturation of DCs
HA	✓		Induction of necroptosis
vRNP	✓		Induction of necroptosis
NA	✓		Induction of apoptosis
GSDME		✓	Induction of pyroptosis and cytokine storm: Stimulation of over‐inflammatory responses
PGE2		✓	Prevention of IFN‐I production
			Reduction of IV antigen presentation to T cells
DDX3X		✓	Activate NLRP3 inflammasome
PPAR‐γ		✓	Suppressive effect on influenza virus upregulate IFN‐I production
ZBP‐1		✓	Prevention of excessive inflammation following IV infection
NLRX1		✓	Prevention of apoptosis induction through interaction with PB1‐F2
GSDMD		✓	Formation of pore in membrane of IV‐infected cells and secretion of IL‐1β and IL‐18 through pyroptosis mechanism

Abbreviations: GSDMD, Gasdermin D; GSDME, Gasdermin E.

## 7 CONCLUSION

Cell death can occur through different mechanisms, including apoptosis, pyroptosis, and necroptosis. These forms of cell death involve specific proteins that contribute to the immune response. For example, DDX3X helps assemble the NLRP3 inflammasome, which has antiviral properties. ZBP1 is involved in regulating pyroptosis and necroptosis and also helps prevent excessive inflammation. PPAR‐γ is found on the surface of AMs and can suppress acute IV infection. On the other hand, PGE2 and GSDME promote inflammation in response to IV infection. Innate immune cells and PANoptosis, which entails various receptors and inflammasomes, effectively eliminate IV infections by creating a pore in the target cell membrane. However, IV can evade the immune response and worsen inflammation through specific proteins such as NS1, PB1‐F2, vRNP, and PA‐X. Treating IV infection with antiviral therapies is crucial, and different signaling pathways can be used in inflammasomes to counteract the virus based on its proteins.

There are different levels of infection caused by IV, with moderate infection resulting in the activation of the PANoptosis mechanism, Inflammasome assembly, and antiviral activity of innate immune cells. This ultimately eliminates the infection. However, when the viral load is high, severe inflammation can occur, which can be lethal. To combat this, antiviral and anti‐inflammatory therapies are required. Corticosteroids are commonly used to treat patients with acute IV infection as they can reduce lung inflammation and other symptoms.^110^ Additionally, type I, and III interferons can suppress the transcription of IL‐1β and inflammasome components. Some studies have shown that certain medications, such as MCC950 (Adipogen), can decrease IV infections by suppressing the NLR3 inflammasome.^111^ Other medications, such as Oridonin, tranilast, OLT1177, Parthenolide, and CY‐09, have also been shown to inhibit the ATPase activity of NLRP3.^112^ However, clinical trials for these medications in IV infection have not yet been conducted. Therefore, it is important to develop specific drugs for anti‐IV therapy that can suppress the excessive activation of inflammasome complexes. Future studies should focus on these drugs.

The interaction and cooperation between three components of the immune system—innate immune cells, inflammasome, and PANoptosis—are impacted by factors such as viral load, influenza strains, immune system health, and spirituality. Despite these variables, a balance is maintained through negative feedback and regulatory molecules to prevent excessive inflammation and limit the spread of infection.

## AUTHOR CONTRIBUTIONS

**Li Wei**: Writing—review & editing. **Xufang Wang**: Writing—original draft. **Huifei Zhou**: Conceptualization; Supervision; Writing—original draft; Writing—review & editing.

## CONFLICT OF INTEREST STATEMENT

The authors declare no conflict of interest.
